# Biomarkers of Host Response Predict Primary End-Point Radiological Pneumonia in Tanzanian Children with Clinical Pneumonia: A Prospective Cohort Study

**DOI:** 10.1371/journal.pone.0137592

**Published:** 2015-09-14

**Authors:** Laura K. Erdman, Valérie D’Acremont, Kyla Hayford, Nimerta Rajwans, Mary Kilowoko, Esther Kyungu, Philipina Hongoa, Leonor Alamo, David L. Streiner, Blaise Genton, Kevin C. Kain

**Affiliations:** 1 Sandra Rotman Centre for Global Health, University Health Network-Toronto General Hospital, Department of Medicine, University of Toronto, Toronto, Ontario, Canada; 2 Swiss Tropical and Public Health Institute, University of Basel, Basel, Switzerland; 3 Department of Ambulatory Care and Community Medicine, University of Lausanne, Lausanne, Switzerland; 4 Amana Regional Referral Hospital, Dar es Salaam, United Republic of Tanzania; 5 St-Francis Hospital, Ifakara, United Republic of Tanzania; 6 Department of Radiology, University Hospital of Lausanne (CHUV), Lausanne, Switzerland; 7 Department of Psychiatry, University of Toronto, Toronto, Ontario, Canada; 8 Infectious Disease Service, University Hospital, Lausanne, Switzerland; Yale University, UNITED STATES

## Abstract

**Background:**

Diagnosing pediatric pneumonia is challenging in low-resource settings. The World Health Organization (WHO) has defined primary end-point radiological pneumonia for use in epidemiological and vaccine studies. However, radiography requires expertise and is often inaccessible. We hypothesized that plasma biomarkers of inflammation and endothelial activation may be useful surrogates for end-point pneumonia, and may provide insight into its biological significance.

**Methods:**

We studied children with WHO-defined clinical pneumonia (n = 155) within a prospective cohort of 1,005 consecutive febrile children presenting to Tanzanian outpatient clinics. Based on x-ray findings, participants were categorized as primary end-point pneumonia (n = 30), other infiltrates (n = 31), or normal chest x-ray (n = 94). Plasma levels of 7 host response biomarkers at presentation were measured by ELISA. Associations between biomarker levels and radiological findings were assessed by Kruskal-Wallis test and multivariable logistic regression. Biomarker ability to predict radiological findings was evaluated using receiver operating characteristic curve analysis and Classification and Regression Tree analysis.

**Results:**

Compared to children with normal x-ray, children with end-point pneumonia had significantly higher C-reactive protein, procalcitonin and Chitinase 3-like-1, while those with other infiltrates had elevated procalcitonin and von Willebrand Factor and decreased soluble Tie-2 and endoglin. Clinical variables were not predictive of radiological findings. Classification and Regression Tree analysis generated multi-marker models with improved performance over single markers for discriminating between groups. A model based on C-reactive protein and Chitinase 3-like-1 discriminated between end-point pneumonia and non-end-point pneumonia with 93.3% sensitivity (95% confidence interval 76.5–98.8), 80.8% specificity (72.6–87.1), positive likelihood ratio 4.9 (3.4–7.1), negative likelihood ratio 0.083 (0.022–0.32), and misclassification rate 0.20 (standard error 0.038).

**Conclusions:**

In Tanzanian children with WHO-defined clinical pneumonia, combinations of host biomarkers distinguished between end-point pneumonia, other infiltrates, and normal chest x-ray, whereas clinical variables did not. These findings generate pathophysiological hypotheses and may have potential research and clinical utility.

## Introduction

Pneumonia causes an estimated 1.4 million childhood deaths annually [1]. Immunization against *Streptococcus pneumoniae* and *Haemophilus influenzae* type B has significantly decreased pneumonia incidence and mortality [2, 3], and vaccine introduction into low-income countries is ongoing. Epidemiological surveillance and vaccine trials are essential for monitoring progress and allocating resources [4].

A major challenge for this research is the lack of a clear definition of pneumonia. Pathologically, pneumonia is a lower respiratory tract infection causing inflammation in the lung parenchyma; pathogen replication activates epithelial cells, macrophages, and endothelial cells, leading to infiltrates and tissue injury [5, 6]. Clinically, pneumonia can manifest variably. World Health Organization (WHO) diagnostic criteria used in community settings in low-income countries–based on cough, tachypnea, and chest indrawing [7]–are poorly suited for research studies. These criteria are highly sensitive, and their implementation decreased childhood pneumonia mortality by 36% [8]; however, they lack specificity and likely capture many non-pneumonia infections [9]. While microbial identification in lung or pleural aspirates could be considered the gold standard, it is invasive and technically challenging, and blood culture is insensitive [10]. Chest radiography has been regarded as a practical gold standard. It has some caveats: infiltrate patterns do not perfectly discriminate bacterial from viral etiology [11–13], nor self-limited infections from those requiring antibiotics [14]. Also, radiography is often unavailable in low-income countries [15].

In 2005, the WHO convened experts to develop a standardized radiological definition of pneumonia for research use. “Primary end-point pneumonia” was defined as alveolar consolidation and/or pleural effusion, and had good intra-observer agreement [16]. It was predicted to be highly specific (though not necessarily sensitive) for bacterial pneumonia. Indeed, in a PCV9 vaccine trial in the Gambia, the incidence of end-point pneumonia was substantially reduced, while rates of WHO-defined clinical pneumonia were unchanged [17]. End-point pneumonia was also associated with bacteremia, especially pneumococcemia [18]. The biological and clinical significance of end-point pneumonia remains unclear. Data from The Gambia suggest it may represent more severe infection: children with end-point pneumonia had sicker appearance, more difficulty breathing and bronchial breath sounds, and higher mortality than those with non-end-point clinical pneumonia [18]. Whether it correlates with a certain group of pathogens or determines the need for antibiotic treatment is yet undefined.

A surrogate for end-point pneumonia would be helpful to avoid the logistics, radiation, and expertise required for radiography. Clinical variables have not been found to accurately or consistently predict radiological pneumonia based on the WHO definition or others [9, 18–20].

Host response biomarkers are increasingly investigated for diagnosis and management of infectious diseases, including respiratory infections [21, 22]. Inflammatory proteins C-reactive protein (CRP) and procalcitonin (PCT) have been evaluated as markers of radiological pneumonia, mostly in adults; results have been variable [23, 24]. In African children with WHO-defined clinical pneumonia, children with end-point pneumonia had higher plasma levels of CRP and PCT than those without [25, 26], consistent with more extensive inflammation on chest radiograph. However, neither marker was highly accurate for predicting x-ray findings [25].

To further this work, an extended panel of plasma host response biomarkers was evaluated for association with end-point pneumonia among Tanzanian children with community-acquired, WHO-defined clinical pneumonia. Markers of inflammation and endothelial activation were tested to explore biological correlates of end-point pneumonia, and to evaluate their utility for predicting radiological findings.

## Materials and Methods

### Study design and participants

This study was nested within a prospective cohort study investigating causes of fever in children (2 months-10 years) presenting at two district hospital outpatient clinics in Tanzania, as described [27]. Briefly, recruitment occurred at Amana Hospital in Dar es Salaam (urban; April-August 2008) and St-Francis Hospital in Ifakara (rural; June-December 2008). These clinics mainly function as primary care facilities for local children and only a small proportion are referrals from surrounding areas. During the study period, vaccines against *S*. *pneumoniae* and *H*. *influenzae* type B were not included in the Tanzanian immunization schedule.

All consecutive children with axillary temperature ≥38°C at presentation and requiring no immediate lifesaving procedures [28] were assessed for inclusion criteria: 1) first visit for the present illness, 2) fever duration ≤1 week, 3) chief reason for visit not injury/trauma, 4) no antimalarial or antibiotic received during the preceding week, and 5) no severe malnutrition. A clinical examination and questionnaire were administered. Nasal and throat swabs and a venous blood sample (EDTA) were collected in all children, and laboratory testing was performed according to pre-defined algorithms [27]. Plasma was stored at -80°C without freeze-thaw until testing.

Clinical pneumonia was diagnosed based on WHO criteria for primary care facilities: cough or difficult breathing, plus fast breathing (≥50 breaths/minute for 2–12 months of age and ≥40 breaths/minute for >12 months) or chest indrawing [7]. Children with clinical pneumonia underwent chest x-ray (CXR). CXRs were read locally for management purposes. For the study, CXR findings were classified according to the WHO Pneumococcal Trialist Ad Hoc Committee recommendations [10, 16] by a pediatric radiologist in Switzerland, who was trained in WHO interpretation and masked to clinical and laboratory data. Patients with alveolar consolidation and/or pleural effusion were designated as “end-point pneumonia.” Those with non-end-point infiltrates were designated as “other infiltrates” (linear and patchy infiltrates involving both lungs, with peribronchial thickening and multiple areas of atelectasis; minor patchy infiltrates of insufficient magnitude to constitute primary end-point consolidation; and small areas of atelectasis). Children with no abnormalities were designated as “normal CXR.” CXRs of inadequate quality precluding interpretation were designated as “indeterminate.”

### Ethics statement

Written parental or guardian informed consent was obtained for all participants and ethics approval was granted by the Ethical Committee of the National Institute for Medical Research (Tanzania) and the Ethikkommission beider Basel (Switzerland). The study was conducted according to the ethical standards of these committees and the Declaration of Helsinki.

### Biomarker assays

The following markers were assayed using R&D Systems ELISA Duoset kits (dilution factor in parentheses): CRP (1:50,000), Chitinase 3-like-1 (CHI3L1; 1:250), soluble Tie2 receptor (sTie-2; 1:50), endoglin (1:50), and P-selectin (1:50). ELISAs were performed according to the manufacturers’ instructions, with the following changes: assays were performed in a volume of 50 μL/well; plasma samples were incubated overnight at 4°C; and ELISAs were developed using Extravidin®-Alkaline Phosphatase (Sigma, 1:1000 dilution, 45 min incubation) followed by addition of p-Nitrophenyl phosphate substrate (Sigma) and optical density readings at 405 nm. PCT (1:5) was assayed using Ray Biotech kits according to the manufacturer’s instructions. Von Willebrand factor (vWF; 1:250) was assayed as described [29]. Briefly, plates were coated with anti-human vWF antibody (Dako, 1:600), and vWF was detected with horseradish peroxidase-conjugated anti-human vWF (Dako, 1:8000) and tetramethylbenzidine (reactions stopped with H_2_SO_4_ and read at 450 nm). Recombinant vWF (American Diagnostica) was used for the standard curve. Background optical density was subtracted prior to analysis. Samples with optical densities below the lowest detectable standard were assigned the value of that standard. ELISAs were conducted blinded to patient diagnosis.

### Data analysis

Continuous variables were compared using non-parametric Mann-Whitney U tests and Kruskal-Wallis tests with Dunn’s test for multiple comparisons. Categorical variables were compared using Chi-square tests with the Bonferroni correction. Multivariate logistic regression models were developed to estimate the association between a single biomarker and end-point pneumonia (versus each other group) after controlling for observed confounders. Receiver operating characteristic (ROC) curves and area under the ROC curve (AUROCC) statistics were generated by the non-parametric method of Hanley et. al. [30] and discriminatory power was judged by the criteria of Hosmer and Lemeshow [31]. Cut-points were generated using the Youden index: J = max(sensitivity + specificity—1) [32]. Classification and regression tree (CRT) analysis, a non-parametric binary recursive partitioning method [33, 34], was employed to predict radiological findings using biomarkers. All tested biomarkers were entered as independent variables, and as continuous variables unless otherwise stated. A minimum of 10 cases for parent nodes and 5 cases for child nodes was specified. Prior probabilities were obtained from the dataset. Pruning was applied to reduce potential over-fitting. Misclassification costs were systematically varied from 1–5 to generate models with a range of sensitivities for the group of primary interest. Misclassification risks were estimated using cross-validation (10 sample folds). 95% confidence intervals were derived using the clinical calculator on vassarstats.net. Analyses were performed with GraphPad Prism version 4.03 (San Diego, CA), IBM SPSS Statistics v22 (Armonk, NY), and Stata11 (College Station, TX).

## Results

### Association of host biomarkers with radiological findings in children with clinical pneumonia

**Fig 1:**
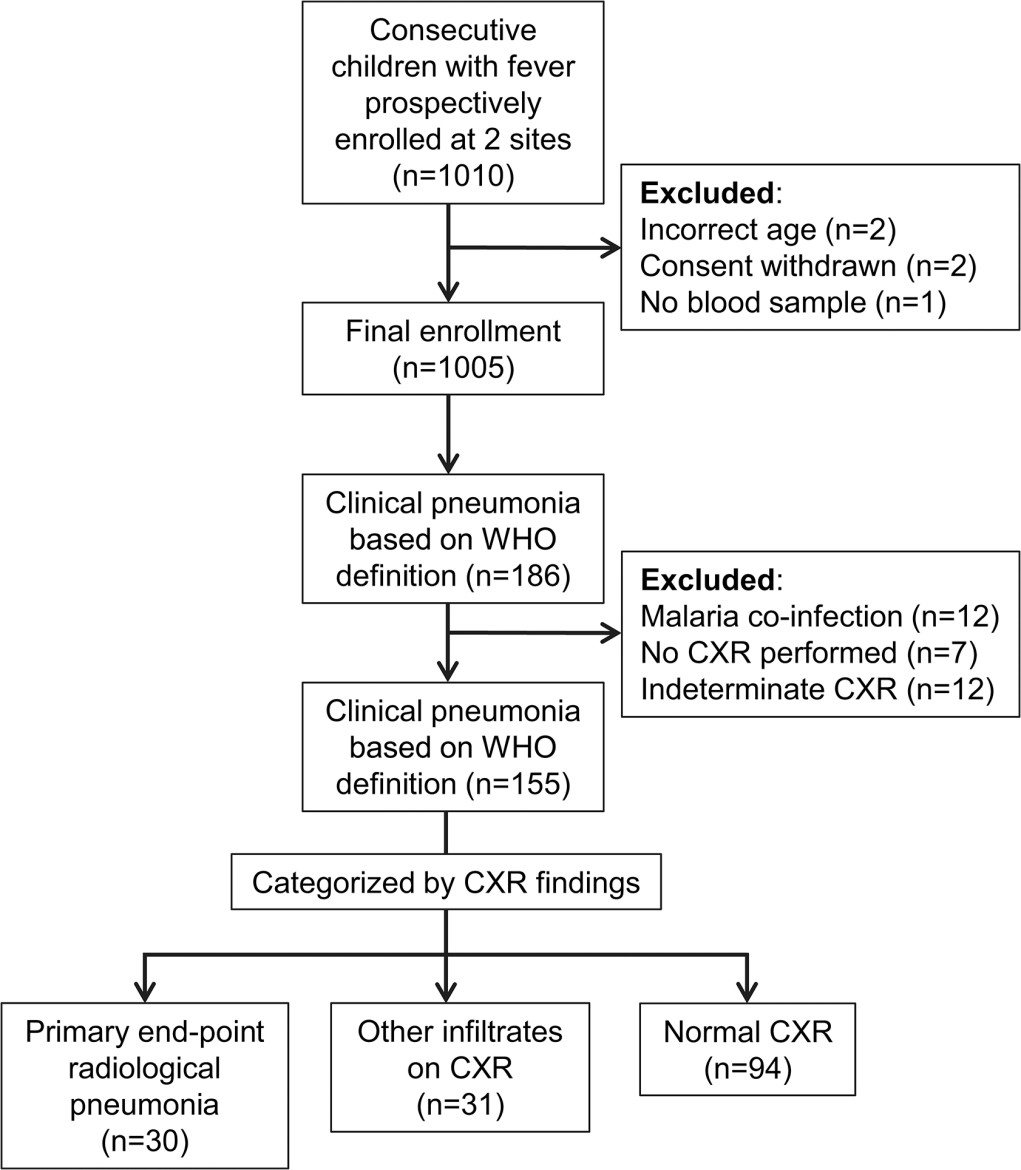
Description of study population. This study was nested in a prospective fever etiology study conducted at the outpatient clinics of two district hospitals in Tanzania. The clinics function as primary care centres for local children. Children presenting with fever were enrolled consecutively, and clinical evaluation and investigations were performed according to pre-defined questionnaires and algorithms. Children with WHO-defined clinical pneumonia were included in the present study, with exclusions as illustrated, and categorized according to findings on chest x-ray (CXR): WHO-defined primary end-point pneumonia, other infiltrates, or normal CXR.

Of 1005 febrile children enrolled in the fever etiology study, 186 children were diagnosed with clinical pneumonia (Fig 1). Children with no CXR performed (n = 7), indeterminate CXR (n = 12), and malaria co-infection (n = 12) were excluded. Thus, a total of 155 children with clinical pneumonia were included in the analysis: 30 with end-point pneumonia, 31 with other infiltrates, and 94 with normal CXR.

Clinically, the three groups were similar, with no significant differences in age, respiratory rate, heart rate, duration of fever, severity, or hospital admission rates (Table 1). Children with end-point pneumonia had higher median temperatures than the other infiltrates group. Only one child (with normal CXR) had a positive blood culture (*Escherichia coli*; cultures only performed on participants recruited in Dar es Salaam). Presence and load of nasopharyngeal carriage of *S*. *pneumoniae* were comparable between groups. Presence of nasopharyngeal viruses was lower in the end-point pneumonia group, but this was not significant. There were no deaths.

**Table 1:** Demographic and clinical characteristics of study participants with WHO-defined clinical pneumonia, categorized by radiological findings.^a^

	End-point pneumonia (n = 30)	Other infiltrates(n = 31)	Normal CXR(n = 94)
Age, months^b^	19.4 [10.7, 36.0]	13.9 [9.3, 28.7]	14.2 [8.8, 24.3]
Gender, number (% female)	19 (63.3)	16 (51.6)	37 (39.4)^#^
Study site, number (% Dar es Salaam)^c^	14 (46.7)	10 (32.3)	35 (37.2)
Severe cases, number (%)^d^	9 (30.0)	7 (22.6)	23 (24.5)
Respiratory rate, breaths/min^e^, 2–12 months (n = 60)	56 [55, 66]	53.5 [52, 56]	56 [53, 60]
Respiratory rate, breaths/min, >12 months (n = 95)	46 [42, 59]	47 [44, 51]	46 [43, 50]
Heart rate, beats/min	130 [107, 150]	126 [111, 147]	124 [108, 148]
Temperature, °C	38.7 [38.2, 39.4]	38.2 [38.0, 38.5]*	38.5 [38.1, 39.0]
Days of fever prior to presentation	2.5 [1.5, 3.0]	2.0 [2.0, 3.0]	3.0 [2.0, 3.0]
Admission to hospital, number (%)	6 (20)	2 (6.5)	7 (7.4)
*Streptococcus pneumoniae* carriage, number (%)	28 (93)	27 (87)	79^f^ (85)
≥1 virus on nasopharyngeal swab, number (%)	23 (77)	26 (84)	84 (89)

Abbreviations: CXR, chest x-ray.

^a^ Kruskal-Wallis test with Dunn’s post-tests used to compare continuous variables; Chi-square test with Bonferroni correction used to compare categorical variables.

*, p<0.05 for other infiltrates versus end-point pneumonia.

^#^, p<0.05 for normal CXR versus end-point pneumonia. Other comparisons were not significantly different.

^b^ All continuous variables had non-normal distributions and are represented as: Median [Interquartile range].

^c^ Study sites were located in Dar es Salaam and Ifakara.

^d^ Severe disease defined according to WHO criteria for the district hospital level.

^e^ Participants were subdivided according to age, as normal values for respiratory rate are age-dependent [7].

^f^ One child in the normal CXR group did not have a *S*. *pneumoniae* swab performed.

**Fig 2:**
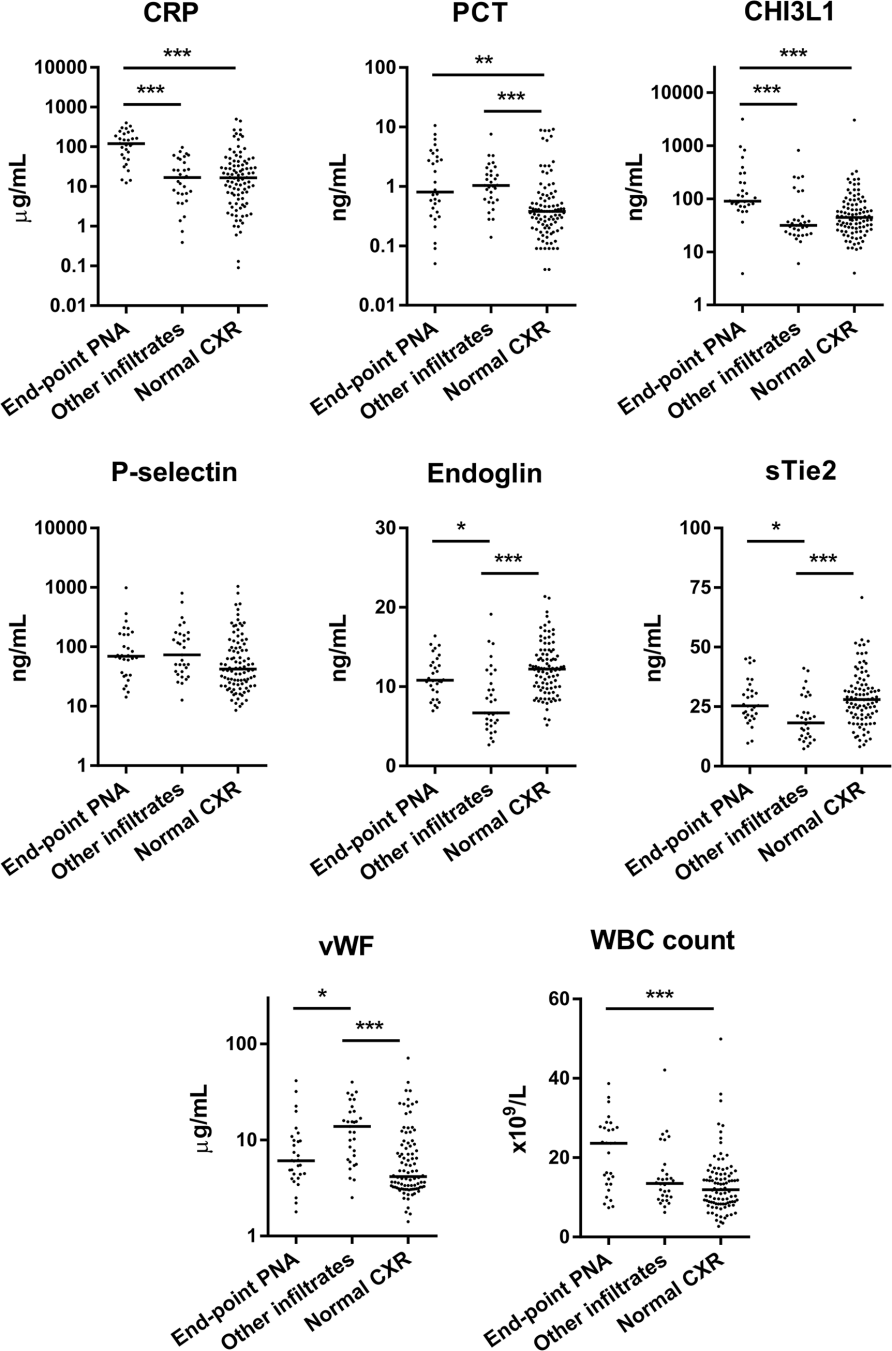
Biomarkers of host response in febrile children with WHO-defined clinical pneumonia, categorized by radiological findings. Plasma collected at presentation was assayed for biomarkers, and biomarker levels were compared between children with end-point pneumonia (End-point PNA; n = 30), other infiltrates (n = 31), and no abnormalities on chest x-ray (Normal CXR; n = 94). * p<0.05, ** p<0.01, and *** p<0.001 by Kruskal-Wallis test with Dunn’s post-tests. All other comparisons were not statistically significant. CHI3L1, Chitinase 3-like-1; CRP, C-reactive protein; CXR, chest x-ray; PCT, procalcitonin; PNA, pneumonia; sTie-2, soluble Tie-2; vWF, von Willebrand Factor; WBC, white blood cell.

Plasma levels of inflammation-associated proteins CRP and CHI3L1 were significantly higher in children with end-point pneumonia compared to the other groups (Fig 2). PCT levels were elevated in both end-point pneumonia and other infiltrates compared to normal CXR. Endothelium-associated markers had a distinct pattern in children with other infiltrates: vWF levels were higher, and sTie-2 and endoglin levels lower, than the other groups. Thus, unique host biomarker profiles were associated with different radiological findings among children with clinical pneumonia, who were otherwise clinically similar.

**Fig 3:**
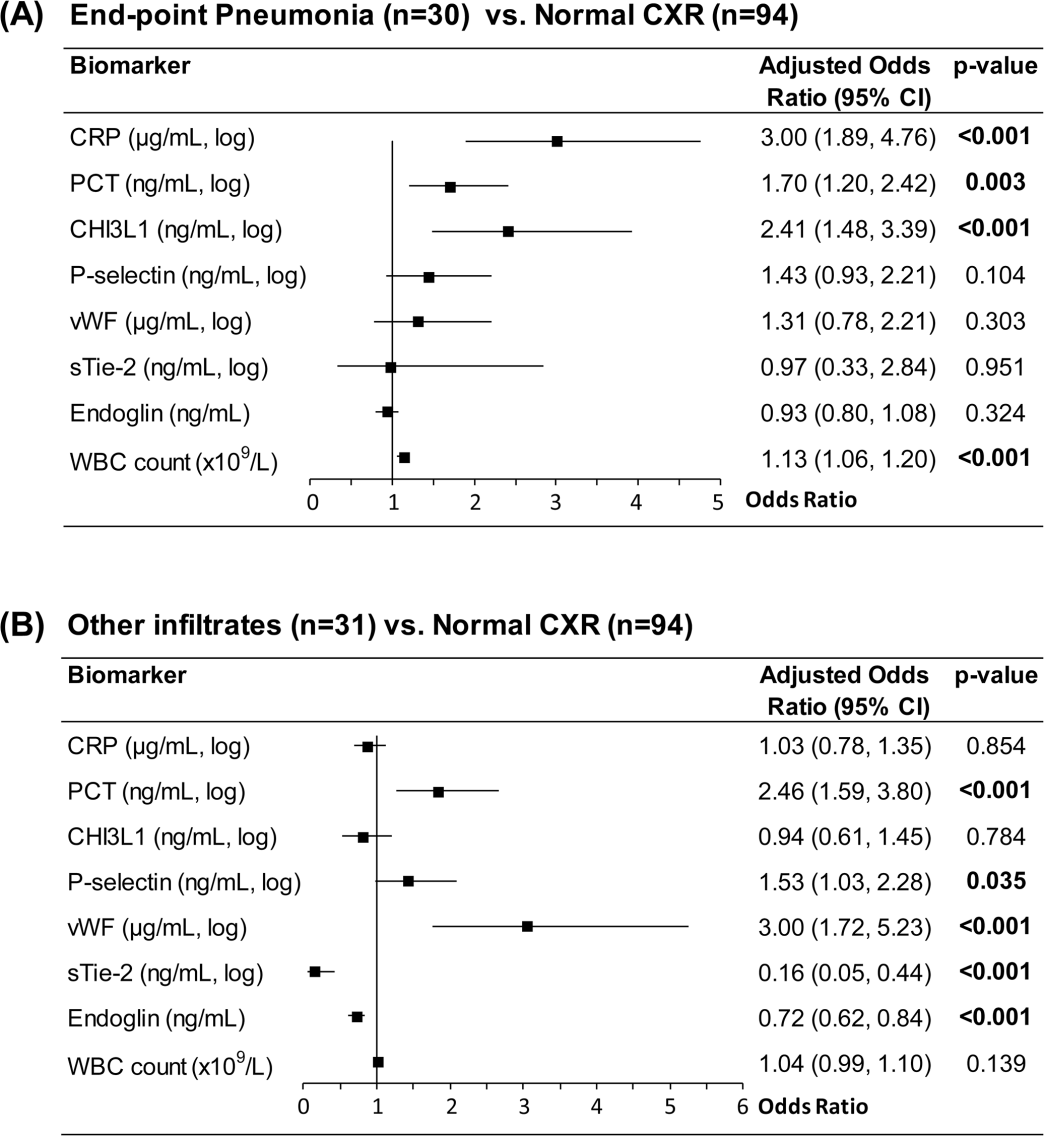
Adjusted associations for biomarkers of host response in children with abnormal versus normal chest x-ray (CXR). Odds ratios were calculated comparing groups using multivariate logistic regression, adjusting for statistically significant demographic and clinical differences between groups. All markers except endoglin and WBC were log transformed since distributions were non-normal. Forest plots show odds ratio point estimates (black squares) and 95% confidence intervals (lines). (A) Odds ratios for end-point pneumonia versus normal CXR, adjusted for age and sex. (B) Odds ratios for other infiltrates versus normal CXR, adjusted for temperature. CHI3L1, Chitinase 3-like-1; CRP, C-reactive protein; CXR, chest x-ray; PCT, procalcitonin; sTie-2, soluble Tie-2; vWF, von Willebrand Factor; WBC, white blood cell.

Multivariate logistic regression models were generated to estimate the degree of association between each biomarker and abnormal CXR findings, after adjusting for clinical and demographic variables significant in bivariate logistic regression (Fig 3). Higher levels of CRP, PCT and CHI3L1 were associated with increased odds of end-point pneumonia compared to normal CXR (Fig 3A). Higher levels of PCT and vWF, and lower levels of sTie-2 and endoglin, were associated with an increased probability of other infiltrates compared to normal CXR (Fig 3B).

White blood cell (WBC) counts in children with end-point pneumonia were increased compared to those with normal CXR (Figs 2 and 3A) as previously described [18]. WBC counts in children with other infiltrates were not significantly different from the other groups (Figs 2 and 3B).

### Biomarker performance for identifying abnormal radiological findings

We assessed the ability of biomarkers to distinguish end-point pneumonia and other infiltrates from normal CXR using ROC curve analysis (AUROCCs in Table 2; ROC curve graphs can be found in S1 Fig). CRP had excellent discrimination between end-point pneumonia and normal CXR, while CHI3L1 and PCT were acceptable. PCT, endoglin, sTie-2, and vWF had acceptable discrimination for other infiltrates versus normal CXR.

**Table 2:** Receiver operating characteristic (ROC) curves and cut-points of biomarkers that significantly discriminate between radiological findings.^a^

Comparison	Biomarker	AUROCC (95% CI)	p value	Cut-point^b^	Sensitivity % (95% CI)	Specificity % (95% CI)	PLR (95% CI)	NLR (95% CI)	PPV % (95% CI)	NPV % (95% CI)
End-point pneumonia versus Normal CXR	CRP	0.85 (0.77, 0.90)	<0.001	>44.1 μg/mL	80.0 (61.4–92.3)	78.7 (69.1–86.5)	3.8 (2.5–5.8)	0.25 (0.1–0.5)	54.5 (38.8–69.6)	92.5 (84.4–97.2)
	PCT	0.70 (0.61, 0.78)	0.001	>0.51 ng/mL	70.0 (50.6–85.3)	69.2 (58.8–78.3)	2.3 (1.5–3.3)	0.43 (0.2–0.8)	42.0 (28.2–56.8)	87.8 (78.2–94.3)
	CHI3L1	0.79 (0.71, 0.86)	<0.001	>56.7 ng/mL	93.3 (77.9–99.2)	62.8 (52.2–72.5)	2.5 (1.9–3.3)	0.11 (0.03–0.4)	44.4 (31.9–57.5)	96.7 (88.6–99.6)
Other infiltrates versus Normal CXR	PCT	0.76 (0.68, 0.83)	<0.001	>0.53 ng/mL	83.9 (66.3–94.5)	71.3 (61.0–80.1)	2.9 (2.0–4.2)	0.23 (0.1–0.5)	49.1 (35.1–63.2)	93.1 (84.5–97.7)
	Endoglin	0.78 (0.70, 0.85)	<0.001	<9.59 ng/mL	71.0 (52.0–85.8)	77.7 (67.9–85.6)	3.2 (2.0–4.9)	0.37 (0.2–0.7)	51.2 (35.5–66.7)	89.0 (80.2–94.9)
	sTie-2	0.72 (0.64, 0.80)	<0.001	<22.6 ng/mL	74.2 (55.4–88.1)	69.2 (58.8–78.3)	2.4 (1.7–3.5)	0.37 (0.2–0.7)	44.2 (30.3–58.8)	89.0 (79.5–95.1)
	vWF	0.77 (0.69, 0.84)	<0.001	>4.8 μg/mL	90.3 (74.2–98.0)	57.5 (46.8–67.6)	2.1 (1.6–2.8)	0.17 (0.06–0.5)	41.2 (29.4–53.8)	94.7 (85.4–98.9)

Abbreviations: AUROCC, area under receiver operating characteristic curve; CHI3L1, Chitinase 3-like-1; CRP, C-reactive protein; CXR, chest x-ray; NLR, negative likelihood ratio; NPV, negative predictive value; PCT, procalcitonin; PLR, positive likelihood ratio; PPV, positive predictive value; ROC, receiver operating characteristic curve; sTie-2, soluble Tie-2; vWF, von Willebrand Factor.

^a^ Children with WHO-defined clinical pneumonia were categorized based on CXR findings: end-point pneumonia, other infiltrates, or normal CXR.

^b^ Cut-points based on Youden index: J = max(sensitivity + specificity – 1).

We examined the predictive ability of single markers using cut-points generated by the Youden Index, which is designed to minimize misclassification rates [32]. For end-point pneumonia, CRP (cut-point >44.1 μg/mL) had the best overall discriminatory ability with 80.0% (95% CI 61.4–92.3) sensitivity and 78.7% (69.1–86.5) specificity (Table 2). For other infiltrates, endoglin (cut-point <9.59 ng/mL) had the best overall discriminatory ability with 71.0% (52.0–85.8) sensitivity and 77.7% (67.9–85.6) specificity (Table 2). Alternative cut-points with >80% sensitivity for detecting the abnormal radiological finding (S1 Table) did not achieve both high sensitivity and specificity.

### Combining biomarkers using Classification and Regression Tree (CRT) analysis improves classification of radiological findings

To see if combining markers improves prediction of CXR findings, we generated decision trees using CRT analysis, a binary recursive partitioning method that optimizes discrimination between groups using an iterative, empiric process. Selected models for end-point pneumonia versus normal CXR are shown in Table 3 (Models 1–3). Model 1 identified end-point pneumonia with 100% (95% CI 85.9–100) sensitivity and 73.4% (63.1–81.7) specificity (S2 Fig). “Pruning” the model to limit over-fitting (Model 2) resulted in 86.7% (68.4–95.6) sensitivity, 79.8% (70.0–86.7) specificity, positive likelihood ratio (PLR) 4.3 (2.8–6.6), and negative likelihood ratio (NLR) 0.17 (0.067–0.42). Compared to single biomarkers (Table 2), CRT models improved sensitivity without sacrificing specificity. Similar results were obtained for other infiltrates versus normal CXR (Table 3, Models 4–6). Thus, combinatorial strategies improved discrimination of children with abnormal versus normal CXRs.

**Table 3:** Classification and Regression Tree (CRT) models classify children with clinical pneumonia based on radiological findings.

Comparison	Model	Specified parameters^a^	Biomarkers in model	Sensitivity % (95% CI)	Specificity % (95% CI)	PLR (95% CI)	NLR (95% CI)	PPV % (95% CI)	NPV % (95% CI)	Misclassification risk (std error)^b^
End-point pneumonia versus Normal CXR	1 (S2 Fig)	Misclassification cost (MC): 3	CRP, Endoglin, P-selectin, sTie2	100 (85.9–100)	73.4 (63.1–81.7)	3.8 (2.7–5.3)	—-	54.5 (40.7–67.8)	100 (93.4–100)	0.28 (0.046)
	2	MC: 3, Pruned	CRP, Endoglin	86.7 (68.4–95.6)	79.8 (70.0–86.7)	4.3 (2.8–6.6)	0.17 (0.067–0.42)	57.8 (42.2–72.0)	95.0 (86.9–98.4)	0.27 (0.047)
	3	MC: 4, Pruned	CHI3L1, CRP	93.3 (76.5–98.8)	72.3 (62.0–80.8)	3.4 (2.4–4.7)	0.092 (0.024–0.35)	51.9 (38.0–65.5)	97.1 (89.1–99.5)	0.29 (0.050)
Other infiltrates versus Normal CXR	4	MC: 2, Pruned	Endoglin, PCT, CHI3L1	80.6 (62.0–91.9)	89.4 (80.9–94.5)	7.6 (4.1–14.0)	0.22 (0.11–0.45)	71.5 (53.5–84.8)	93.3 (85.5–97.3)	0.21 (0.040)
	5	MC: 3, Pruned	PCT, vWF, CRP	93.5 (77.2–98.9)	74.5 (64.3–82.7)	3.7 (2.6–5.2)	0.087 (0.023–0.33)	54.7 (40.6–68.2)	97.2 (89.4–99.5)	0.32 (0.046)
	6	MC: 4, Pruned	PCT, CRP	83.9 (65.5–93.9)	79.8 (70.0–87.1)	4.2 (2.7–6.4)	0.20 (0.090–0.45)	57.8 (42.2–72.0)	93.8 (85.4–97.7)	0.20 (0.044)
End-point pneumonia versus Non-end-point pneumonia^d^	7	MC: 2, Pruned	CRP, CHI3L1x2^c^	86.7 (68.4–95.6)	87.2 (79.8–92.3)	6.8 (4.2–10.9)	0.15 (0.061–0.38)	61.9 (45.7–76.0)	96.5 (90.6–98.9)	0.24 (0.037)
	8	MC: 4, Pruned	CHI3L1, CRP	93.3 (76.5–98.8)	76.0 (67.4–83.0)	3.9 (2.8–5.4)	0.088 (0.23–0.34)	48.3 (35.1–61.7)	97.9 (92.0–99.6)	0.24 (0.044)
	9(Fig 4)	MC: 4, Pruned, CRP/PCT preset^e^	CRP, CHI3L1x2	93.3 (76.5–98.8)	80.8 (72.6–87.1)	4.9 (3.4–7.1)	0.083 (0.022–0.32)	53.8 (39.6–67.5)	98.1 (92.5–99.7)	0.20 (0.038)

Abbreviations: CHI3L1, Chitinase 3-like-1; CRP, C-reactive protein; CRT, Classification and Regression Tree; CXR, chest x-ray; MC, misclassification cost; NLR, negative likelihood ratio; NPV, negative predictive value; PCT, procalcitonin; PLR, positive likelihood ratio; PPV, positive predictive value; sTie-2, soluble Tie-2; vWF, von Willebrand Factor.

^a^ Minimum number of cases was 10 per parent node (prior to split) and 5 per child node (following split). Misclassification cost is how many fold worse it is to misclassify the group of interest versus the other group. Pruning reduces overfitting by trimming trees down to simpler structures.

^b^ Generated using cross-validation with 10 sample folds.

^c^ x2 = model contains 2 different cut-points for a single biomarker.

^d^ Other infiltrates and normal CXR groups were combined into “non-end-point pneumonia.”

^e^ CRP and PCT were entered as categorical variables based on cut-points used in available point-of-care tests: CRP 40 μg/mL and PCT 0.5 ng/mL.

**Fig 4:**
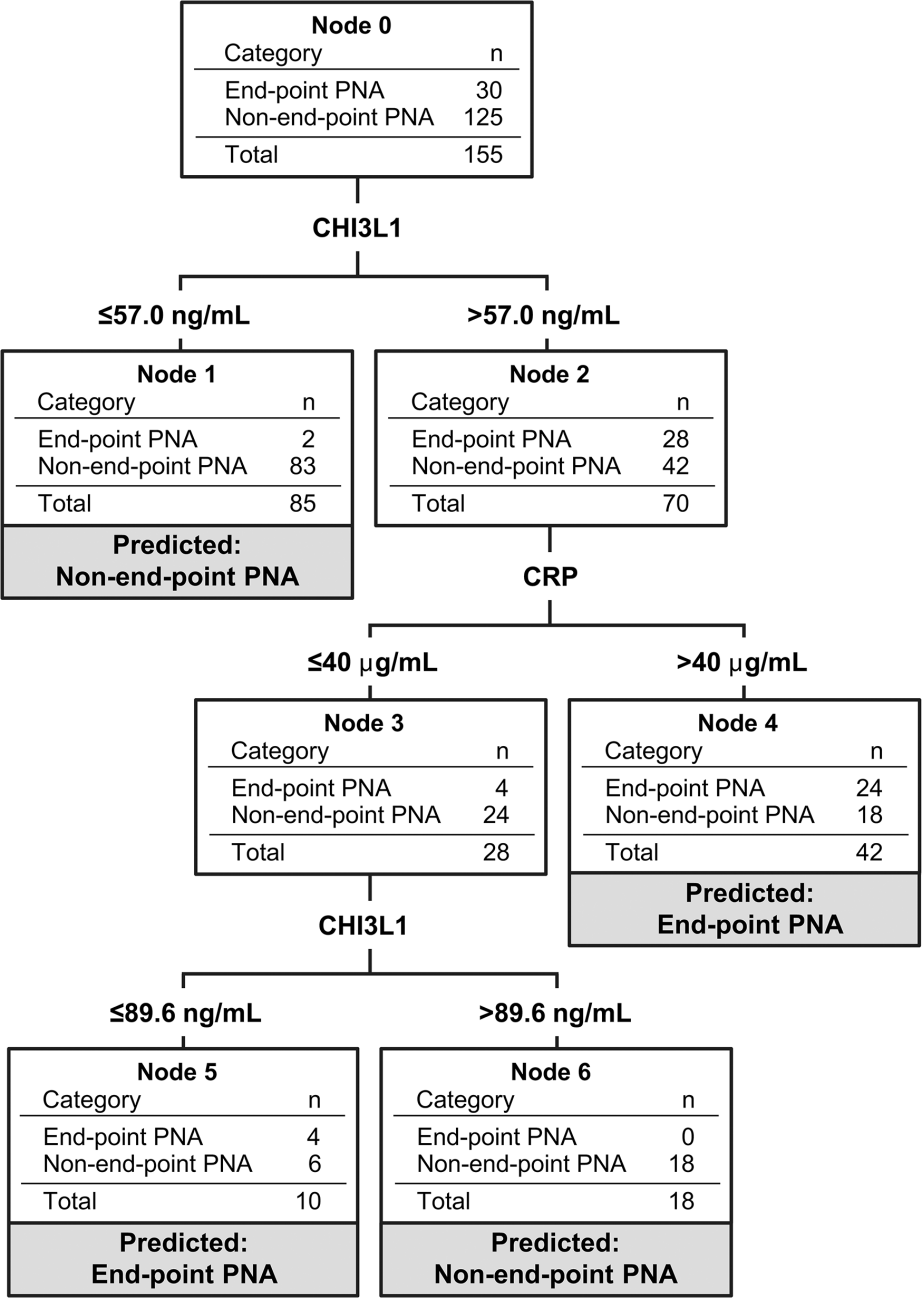
Classification and Regression Tree model uses biomarkers to discriminate between end-point pneumonia and non-end-point pneumonia. Classification and Regression Tree (CRT) modelling was used to improve upon performance of single biomarkers for distinguishing between end-point pneumonia and non-end-point pneumonia (comprising “other infiltrates” and “normal chest x-ray (CXR)” groups combined). All 7 biomarkers were entered into the CRT analysis as independent variables. Minimum number of cases was designated as 10 for parent nodes (prior to split) and 5 for child nodes (following split). Children in terminal nodes of the tree were classified into the category indicated (i.e., “Predicted:…”). Shown here is a representation of Model 9 in Table 3. For this model, cut-points were pre-specified for CRP (40 μg/mL) and PCT (0.5 ng/mL) based on commercially available tests. Performance characteristics were as follows: sensitivity 93.3% (76.5–98.8), specificity 80.8% (72.6–87.1), positive likelihood ratio 4.9 (3.4–7.1), negative likelihood ratio 0.083 (0.022–0.32), positive predictive value 53.8% (39.6–67.5), negative predictive value 98.1% (92.5–99.7), misclassification risk 0.20 (standard error 0.038). CHI3L1, Chitinase 3-like-1; CRP, C-reactive protein; CRT, Classification and Regression Tree; CXR, chest x-ray; PCT, procalcitonin; PNA, pneumonia.

Since end-point pneumonia is used as an outcome in epidemiological and vaccine studies, we repeated the analysis comparing children with end-point pneumonia (n = 30) to those with non-end-point pneumonia (other infiltrates and normal CXR combined, n = 125). The two groups were clinically similar, except that the end-point pneumonia group was significantly older with more females and more hospital admissions (S2 Table). Plasma levels of CRP, PCT, and CHI3L1 were elevated in end-point pneumonia compared to non-end-point pneumonia and significant in ROC curve analysis (S3 Fig and S3 Table). CRT analysis identified models with sensitivities >85% and specificities >75% (Table 3, Models 7–9). When CRP and PCT were dichotomized based on cut-points of existing point-of-care tests, a model based on CRP and CHI3L1 with 93.3% (76.5–98.8) sensitivity, 80.8% (72.6–87.1) specificity, PLR 4.9 (3.4–7.1), NLR 0.083 (0.022–0.32), and misclassification rate 0.20 (standard error 0.038) was generated (Model 9; Fig 4).

## Discussion

In this study of Tanzanian pediatric outpatients with clinical pneumonia, we show that distinct patterns of host response biomarkers were associated with end-point pneumonia, other infiltrates, and normal CXR, while clinical variables were similar between groups. CRT analysis generated biomarker combinations with improved accuracy for predicting end-point pneumonia over single markers, with potential application in community-based research and clinical settings. These data also suggest that radiological findings may reflect meaningful pathophysiological differences between respiratory infections.

Clinical features were poor predictors of radiographic findings, similar to a study of non-severe pediatric pneumonia in Pakistan [9] and a recent systematic review [20]. In contrast, there was increased disease severity and mortality among children with end-point versus non-end-point pneumonia in The Gambia [18]. The Gambian study included children with life-threatening illness and malnutrition, while our study did not. Interestingly, respiratory rate did not differ between groups in our study. Factors other than alveolar consolidation can cause tachypnea, such as fever, upper airway congestion, or acidosis.

Despite clinical similarities, inflammatory biomarkers were significantly elevated in children with end-point pneumonia compared to normal CXR. CRP and PCT are acute phase proteins induced by inflammation. Our results confirm previous reports showing increased CRP and PCT with radiological consolidation [25, 26]. AUROCCs and cut-points in our study are similar to those in Gambian children [25], suggesting consistency across African settings. In another Gambian study, CRP, lipocalin-2 and vWF were associated with pediatric bacterial pneumonia using a composite diagnosis based on radiography, blood culture, and WBC count [35]. Different definitions of pneumonia may explain the discordant vWF results.

We identified CHI3L1 as a novel marker in pediatric pneumonia. CHI3L1 is a soluble glycoprotein induced by cytokines and injurious stimuli [36]. In adults, serum CHI3L1 levels were elevated in streptococcal pneumonia compared to pneumonia of unknown etiology and healthy controls [37]. In a murine model, streptococcal pneumonia induced CHI3L1, and *Chi3l1* deletion led to macrophage dysfunction, increased bacterial loads, and reduced survival [38]. Whether CHI3L1 confers protection in human pneumonia warrants further investigation.

The “other infiltrates” group had a unique biomarker profile, with alterations in endothelial activation markers and few changes in inflammatory markers compared to normal CXR. Given the young median age and infiltrate pattern, many likely had viral bronchiolitis. Viral infection of bronchiolar epithelium [39] may not activate systemic inflammatory responses as intensely as alveolar infections. Respiratory syncytial virus, a major cause of bronchiolitis and the most common virus isolated in the “other infiltrates” group, infects and activates human endothelial cells in vitro [40], possibly contributing to the observed endothelial marker profile. The extent and effects of endothelial activation in bronchiolitis could be explored in mechanistic studies.

As CXR patterns are not strictly correlated with causal pathogens [11–13], some children with “other infiltrates” may have had bacterial pneumonia rather than viral bronchiolitis. Similarly, end-point pneumonia was likely caused by a variety of organisms. This etiological diversity and host factors probably contributed to intra-group biomarker variability. Nonetheless, distinct biomarker profiles were associated with each radiological pattern, indicating some convergence of host response pathways. Defining these pathways may identify therapeutic targets in respiratory infections involving host immunopathology, regardless of the pathogen [5, 39].

From a practical perspective, predicting end-point pneumonia using host biomarkers would be useful for epidemiological and vaccine research. We provide proof-of-principle that CRT analysis can generate combinatorial models with improved predictive accuracy over individual biomarkers. Some markers included in CRT models (e.g., P-selectin, vWF) were not informative in bivariate analysis. This may reflect interactions between pathways, and highlights the value of unbiased approaches [21, 41]. If suitable models are identified and validated, advances in point-of-care platforms for rapid, inexpensive analysis of multiple markers in small blood volumes [42] could make multiplex biomarker testing feasible in resource-constrained research settings.

Biomarker prediction of radiological findings may also inform clinical decision-making. It is difficult to know how accurately x-ray distinguishes bacterial from viral respiratory infections, given the technical challenges of determining microbial etiology [10, 43]. We postulate that, in our immunocompetent population, the nature and intensity of host responses–as reflected by radiographic findings and biomarker profiles–can help guide antibiotic treatment. Children with normal chest x-rays typically had low levels of inflammatory markers, suggesting upper respiratory infection, which was likely viral. However, all received antibiotics according to WHO guidelines. Such treatment may lead to unnecessary costs, side effects, and antimicrobial resistance. Conversely, end-point pneumonia was characterized by elevated systemic inflammatory markers, indicating activation of the immune system. This is more likely to represent bacterial infection and warrants treatment. Such an approach may lead to overtreatment of viral infections causing consolidation; as these cases are prone to secondary bacterial pneumonia [44], antibiotics may in fact be clinically justified.

Whether “other infiltrates” require antimicrobial treatment is unclear. In a randomized trial in Pakistan, there was no difference in outcomes among children <5 years with WHO-defined non-severe clinical pneumonia treated with amoxicillin or placebo [45]; 12% of this population had interstitial infiltrates based on prior data (only 1.4% had end-point pneumonia) [9]. This suggests that in this age group, interstitial infiltrates may represent viral infections or self-resolving bacterial infections. If this result is confirmed, a biomarker test for end-point pneumonia could identify children most likely to benefit from antibiotics. Ideally, to provide maximal information, biomarker models could separate children into each of the 3 radiological outcomes. This is possible with CRT analysis, but would require a larger dataset.

In high-income countries, PCT has been investigated for guiding antibiotic treatment in lower respiratory tract infections, and this strategy reduced antibiotic use with no difference in adverse outcomes [14, 46, 47]. Prospective pediatric trials in low-resource settings could examine the value of biomarker surrogates of radiological findings to inform treatment decisions. Of course, any such approach should incorporate clinical judgment: children with clinically severe illness need treatment regardless of investigation results, and families of children with non-severe non-end-point pneumonia who are not given antibiotics require counselling regarding danger signs.

Limitations of this study include sample size, a single CXR reader, and inability to statistically compare models due to the empiric basis of CRT analysis. Only children febrile at presentation were recruited, but fever can be intermittent or obscured by anti-pyretic use, and it is not presently required for WHO-defined clinical pneumonia. Critically ill children were excluded, which may have disproportionately eliminated end-point pneumonia cases due to association with severe disease [18]. Malaria co-infections were excluded because malaria affects biomarker levels [25, 48] and stratified analysis was not possible due to small numbers of malaria infections. Biomarker models require external validation in populations with a broader spectrum of disease severity, co-infection, and pneumonia epidemiology. Future studies should evaluate how pneumonia vaccine introduction may affect test characteristics (e.g., positive predictive value).

Strengths of this study include a consecutive design, use of clinically relevant WHO definitions, and a low-resource community setting, which encompasses the majority of childhood pneumonia cases globally. The CRT-based combinatorial approach is novel in this field. Pending validation, these findings may form the basis of a surrogate test to identify end-point pneumonia, with potential application in epidemiological studies and clinical work.

## Supporting Information

S1 Dataset Demographic, clinical, and biomarker data. (XLSX)

S1 Fig Receiver operator characteristic (ROC) curves for biomarkers that discriminate between radiological findings. ROC curves were generated by the non-parametric method of Hanley et. al. and are shown for (A) end-point pneumonia versus normal chest x-ray (CXR) and (B) other infiltrates versus normal CXR. The dashed reference line represents the ROC curve for a test with no discriminatory ability. Area under the curve and p values are displayed in Table 2. CHI3L1, Chitinase 3-like-1; CRP, C-reactive protein; CXR, chest x-ray; PCT, procalcitonin; sTie-2, soluble Tie-2; vWF, von Willebrand Factor.
(TIF)

S2 Fig Classification and Regression Tree (CRT) model that uses biomarkers to discriminate between children with end-point pneumonia and normal CXR. CRT modelling was used to improve upon performance of single biomarkers for distinguishing between end-point pneumonia and normal chest x-ray (CXR). All 7 biomarkers were entered into the CRT analysis as independent variables. Minimum number of cases was designated as 10 for parent nodes (prior to split) and 5 for child nodes (following split). Children in terminal nodes of the tree were classified into the category indicated (i.e., “Predicted:…”). Shown here is a representation of Model 1 in Table 3. Performance characteristics were as follows: sensitivity 100% (85.9–100), specificity 73.4% (63.1–81.7), positive likelihood ratio 3.8 (2.7–5.3), negative likelihood ratio–not applicable, positive predictive value 54.5% (40.7–67.8), negative predictive value 100% (93.4–100), misclassification risk 0.28 (standard error 0.046). CRP, C-reactive protein; CXR, chest x-ray; PNA, pneumonia; sTie-2, soluble Tie-2.
(TIF)

S3 Fig Comparison of host response biomarkers in clinical pneumonia cases with and without primary end-point pneumonia. (A) Plasma collected at time of presentation was assayed for biomarkers, and biomarker levels were compared between children with end-point pneumonia (End-point PNA; n = 30), and those with either other infiltrates or normal chest x-ray (Non-end-point PNA; n = 125). * p<0.05 and *** p<0.001 by Kruskal-Wallis test with Dunn’s post-tests. All other comparisons were not statistically significant. (B) Receiver operating characteristic curves are shown for biomarkers that significantly discriminated between the two groups. Area under the curve and cut-points are displayed in S3 Table. CHI3L1, Chitinase 3-like-1; CRP, C-reactive protein; CXR, chest x-ray; PCT, procalcitonin; PNA, pneumonia; sTie-2, soluble Tie-2; vWF, von Willebrand Factor; WBC, white blood cell.
(TIF)

S1 Table Alternative cut-points for biomarkers that discriminate between clinical pneumonia cases with and without radiological abnormalities, with >80% sensitivity for the group with chest x-ray findings. (DOCX)

S2 Table Demographic and clinical characteristics of study participants with WHO-defined clinical pneumonia with and without end-point pneumonia on chest x-ray. (DOCX)

S3 Table Receiver operating characteristic (ROC) curves and cut-points of biomarkers that significantly discriminate between clinical pneumonia cases with (n = 30) and without (n = 125) end-point pneumonia on chest x-ray. (DOCX)
