# Supplementary figures and images for: Biomarkers of Host Response Predict Primary End-Point Radiological Pneumonia in Tanzanian Children with Clinical Pneumonia: A Prospective Cohort Study
Source: PLoS One. 2015 Sep 14;10(9):e0137592. doi: 10.1371/journal.pone.0137592 (PMC4569067; doi:10.1371/journal.pone.0137592)

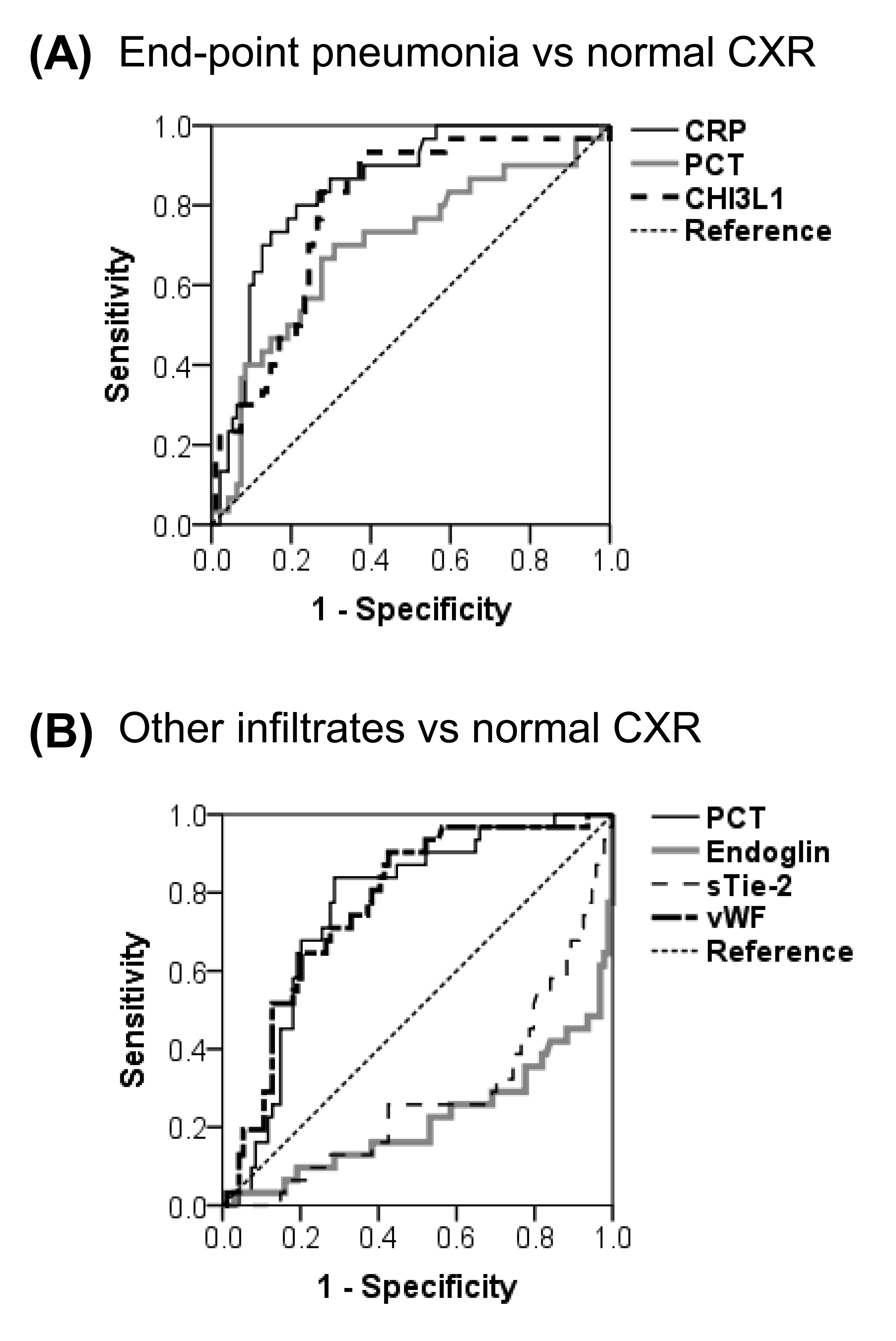

Supplement: S1 Fig — ROC curves were generated by the non-parametric method of Hanley et. al. and are shown for (A) end-point pneumonia versus normal chest x-ray (CXR) and (B) other infiltrates versus normal CXR. The dashed reference line represents the ROC curve for a test with no discriminatory ability. Area under the curve and p values are displayed in Table 2. CHI3L1, Chitinase 3-like-1; CRP, C-reactive protein; CXR, chest x-ray; PCT, procalcitonin; sTie-2, soluble Tie-2; vWF, von Willebrand Factor. (TIF) [file pone.0137592.s002.tif]

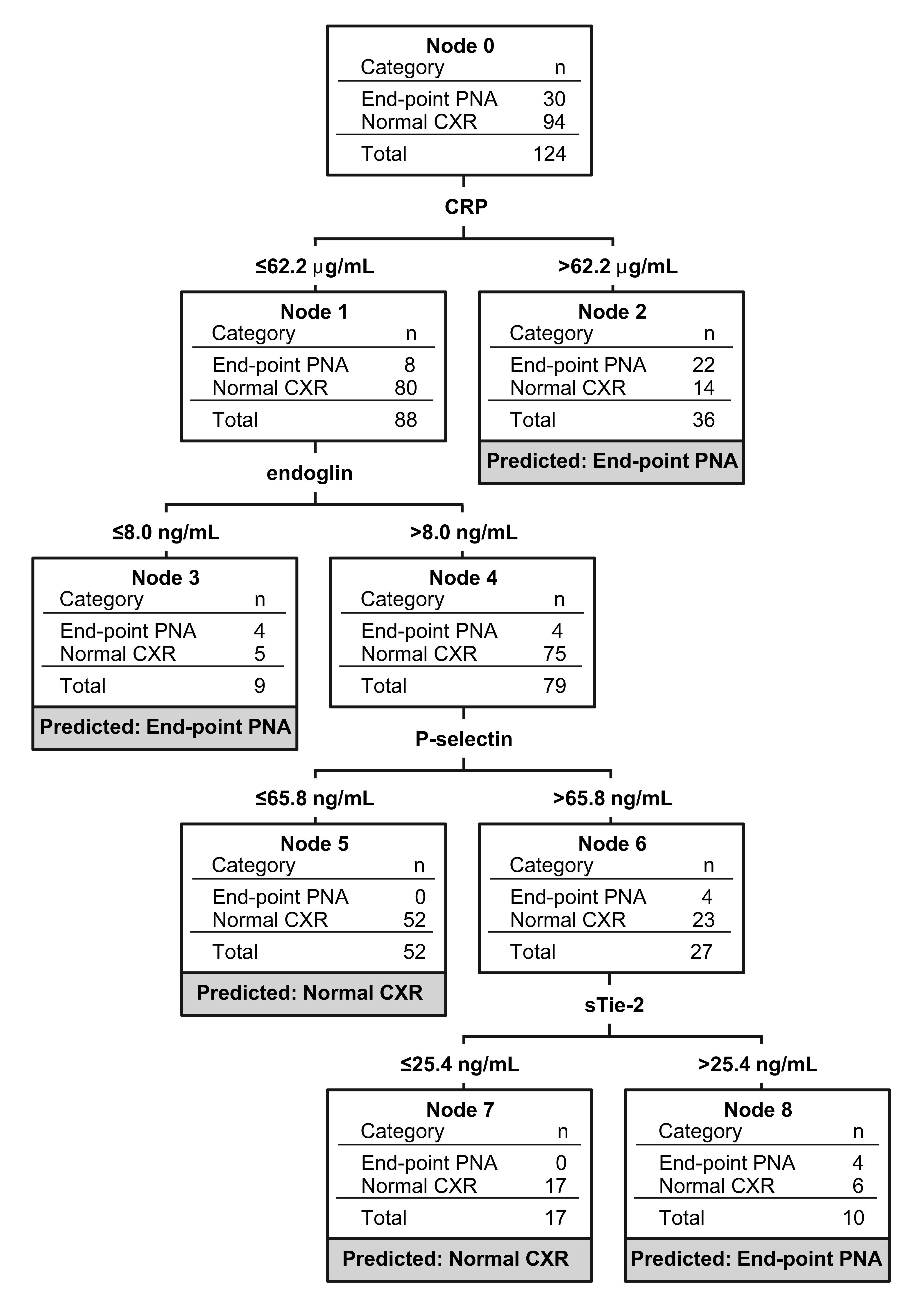

Supplement: S2 Fig — CRT modelling was used to improve upon performance of single biomarkers for distinguishing between end-point pneumonia and normal chest x-ray (CXR). All 7 biomarkers were entered into the CRT analysis as independent variables. Minimum number of cases was designated as 10 for parent nodes (prior to split) and 5 for child nodes (following split). Children in terminal nodes of the tree were classified into the category indicated (i.e., “Predicted:…”). Shown here is a representation of Model 1 in Table 3. Performance characteristics were as follows: sensitivity 100% (85.9–100), specificity 73.4% (63.1–81.7), positive likelihood ratio 3.8 (2.7–5.3), negative likelihood ratio–not applicable, positive predictive value 54.5% (40.7–67.8), negative predictive value 100% (93.4–100), misclassification risk 0.28 (standard error 0.046). CRP, C-reactive protein; CXR, chest x-ray; PNA, pneumonia; sTie-2, soluble Tie-2. (TIF) [file pone.0137592.s003.tif]

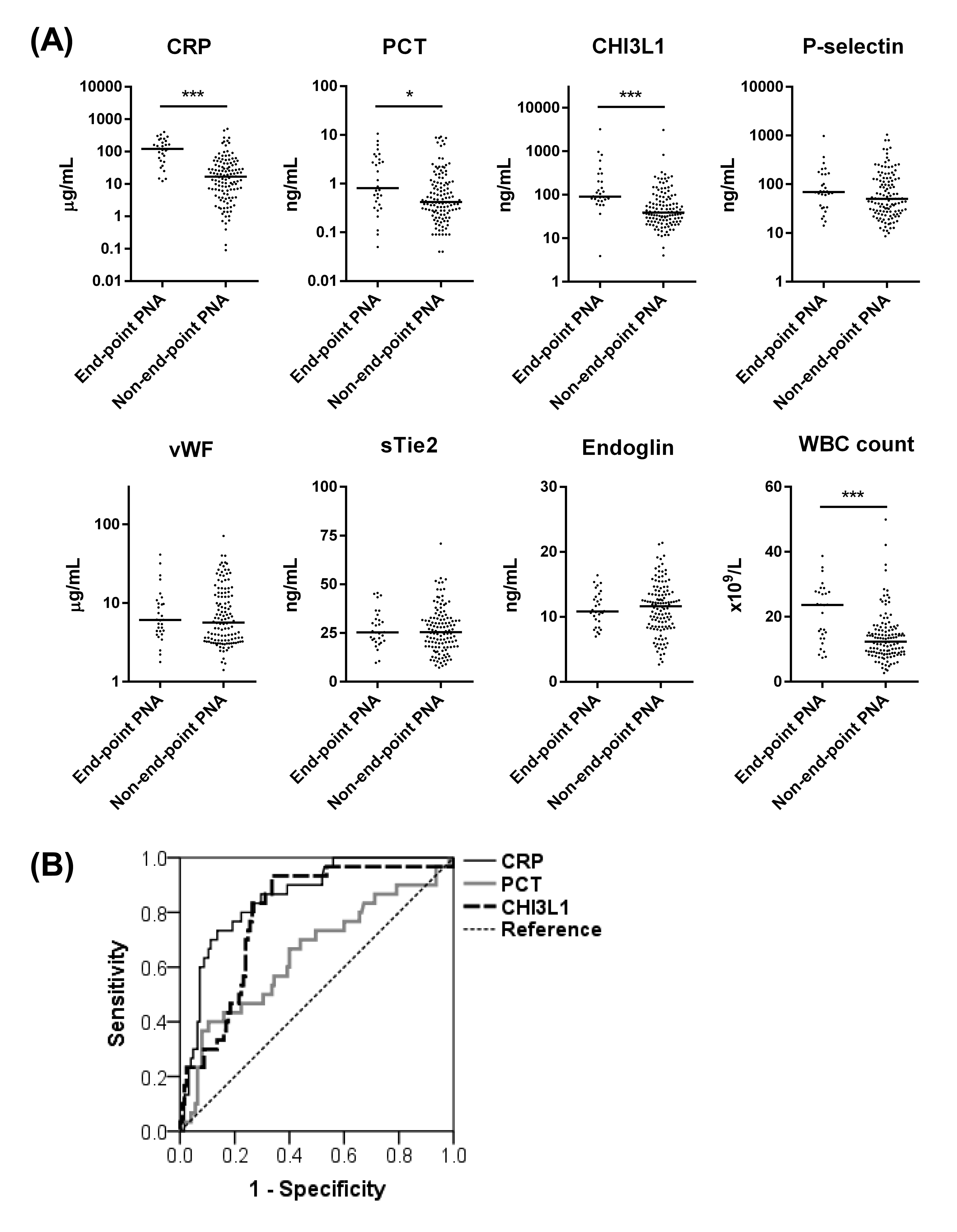

Supplement: S3 Fig — (A) Plasma collected at time of presentation was assayed for biomarkers, and biomarker levels were compared between children with end-point pneumonia (End-point PNA; n = 30), and those with either other infiltrates or normal chest x-ray (Non-end-point PNA; n = 125). * p<0.05 and *** p<0.001 by Kruskal-Wallis test with Dunn’s post-tests. All other comparisons were not statistically significant. (B) Receiver operating characteristic curves are shown for biomarkers that significantly discriminated between the two groups. Area under the curve and cut-points are displayed in S3 Table. CHI3L1, Chitinase 3-like-1; CRP, C-reactive protein; CXR, chest x-ray; PCT, procalcitonin; PNA, pneumonia; sTie-2, soluble Tie-2; vWF, von Willebrand Factor; WBC, white blood cell. (TIF) [file pone.0137592.s004.tif]
