# Supplementary material for: Biomarkers of Host Response Predict Primary End-Point Radiological Pneumonia in Tanzanian Children with Clinical Pneumonia: A Prospective Cohort Study
Source: PLoS One. 2015 Sep 14;10(9):e0137592. doi: 10.1371/journal.pone.0137592 (PMC4569067; doi:10.1371/journal.pone.0137592)
Supplement: S1 Table — (DOCX) [file pone.0137592.s005.docx]

**S1 Table. Alternative cut-points for biomarkers that discriminate between clinical pneumonia cases with and without radiological abnormalities,^a^ with >80% sensitivity for the group with chest x-ray findings.**

| **Comparison** | **Marker** | **Cut-point** | **Sensitivity %**  **(95% CI)** | **Specificity %**  **(95% CI)** | **PLR**  **(95% CI)** | **NLR**  **(95% CI)** | **PPV %**  **(95% CI)** | **NPV %**  **(95% CI)** |
| --- | --- | --- | --- | --- | --- | --- | --- | --- |
| End-point  Pneumonia  Versus  Normal CXR | **CRP** (μg/mL) | >29.8 | 86.7 (69.3-96.2) | 70.2 (59.9-79.2) | 2.9 (2.1-4.1) | 0.19 (0.08-0.5) | 48.1 (34.3-62.2) | 94.3 (86.0-98.4) |
|  |  | >24.3 | 90.0 (73.5-97.9) | 61.7 (51.1-71.5) | 2.4 (1.8-3.1) | 0.16 (0.05-0.5) | 42.9 (30.5-56.0) | 95.1 (86.3-99.0) |
|  |  | >13.5 | 96.7 (82.8-99.9) | 46.7 (36.4-57.4) | 1.8 (1.5-2.2) | 0.071 (0.01-0.5) | 36.7 (26.1-48.3) | 97.8 (88.2-99.9) |
|  | **PCT** (ng/mL) | >0.32 | 80.0 (61.4-92.3) | 42.6 (32.4-53.2) | 1.4 (1.1-1.8) | 0.47 (0.2-1.0) | 30.8 (20.8-42.2) | 87.0 (73.7-95.1) |
|  | **CHI3L1**  (ng/mL) | >70.6 | 80.0 (61.4-92.3) | 72.3 (62.2-81.1) | 2.9 (2.0-4.2) | 0.28 (0.1-0.6) | 48.0 (33.7-62.6) | 91.9 (83.2-97.0) |
|  |  | >59.6 | 86.7 (69.3-96.2) | 66.0 (55.5-75.4) | 2.6 (1.9-3.5) | 0.20 (0.08-0.5) | 44.8 (31.7-58.5) | 93.9 (85.1-98.3) |
|  |  | >35.2 | 96.7 (82.8-99.9) | 41.5 (31.4-52.1) | 1.7 (1.4-2.0) | 0.080 (0.01-0.6) | 34.5 (24.5-45.7) | 97.5 (86.8-99.9) |

| Other infiltrates  Versus  Normal CXR | **PCT** (ng/mL) | >0.36 | 90.3 (74.2-98.0) | 47.9 (37.5-58.4) | 1.7 (1.4-2.2) | 0.20 (0.07-0.6) | 36.4 (25.7-48.1) | 93.7 (82.8-98.7) |
| --- | --- | --- | --- | --- | --- | --- | --- | --- |
|  | **Endoglin** (ng/mL) | <12.1 | 80.7 (62.5-92.5) | 53.2 (42.6-63.6) | 1.7 (1.3-2.3) | 0.36 (0.2-0.8) | 36.2 (25.0-48.7) | 89.3 (78.0-96.0) |
|  | **sTie-2** (ng/mL) | <29.9 | 80.7 (62.5-92.5) | 42.6 (32.4-53.2) | 1.4 (1.1-1.8) | 0.45 (0.2-1.0) | 31.6 (21.6-43.1) | 87.0 (73.7-95.1) |
|  | **vWF** (μg/mL) | >5.8 | 80.7 (62.5-92.5) | 61.7 (51.1-71.5) | 2.1 (1.5-2.9) | 0.31 (0.2-0.7) | 41.0 (28.6-54.3) | 90.6 (80.7-96.5) |
|  |  | >5.4 | 87.1 (70.2-96.4) | 58.5 (47.9-68.6) | 2.1 (1.6-2.8) | 0.22 (0.09-0.6) | 40.9 (29.0-53.7) | 93.2 (83.5-98.1) |
|  |  | >4.0 | 93.6 (78.6-99.2) | 47.9 (37.5-58.4) | 1.8 (1.4-2.2) | 0.13 (0.03-0.5) | 37.2 (26.4-48.9) | 95.7 (85.5-99.5) |

Abbreviations: CHI3L1, Chitinase 3-like-1; CRP, C-reactive protein; CXR, chest x-ray; NLR, negative likelihood ratio; NPV, negative predictive value; PCT, procalcitonin; PLR, positive likelihood ratio; PPV, positive predictive value; sTie-2, soluble Tie-2; vWF, von Willebrand Factor.

^a^ Children with clinical pneumonia were categorized based on chest x-ray findings: end-point pneumonia, other infiltrates, or normal CXR.
