# Supplementary material for: Biomarkers of Host Response Predict Primary End-Point Radiological Pneumonia in Tanzanian Children with Clinical Pneumonia: A Prospective Cohort Study
Source: PLoS One. 2015 Sep 14;10(9):e0137592. doi: 10.1371/journal.pone.0137592 (PMC4569067; doi:10.1371/journal.pone.0137592)
Supplement: S2 Table — (DOCX) [file pone.0137592.s006.docx]

**S2 Table. Demographic and clinical characteristics of study participants with WHO-defined clinical pneumonia with and without end-point pneumonia on chest x-ray.^a^**

|  | **End-point pneumonia**  **(n=30)** | **Non-end-point pneumonia**  **(n=125)** |
| --- | --- | --- |
| Age, months^b^ | 19.4 [10.7, 36.0] | 14.1 [9.1, 25.0]* |
| Gender, number (% female) | 19 (63.3) | 53 (42.4)* |
| Study site, number (% Dar es Salaam)^c^ | 14 (46.7) | 45 (36.0) |
| Severe cases, number (%)^d^ | 9 (30.0) | 30 (24.0) |
| Respiratory rate, breaths/min^e^, 2-12 months (n=60) | 56 [55, 66] | 56 [53, 59] |
| Respiratory rate, breaths/min, >12 months (n=95) | 46 [42, 59] | 47 [43, 51] |
| Heart rate, beats/min | 130 [107, 150] | 125 [109, 147] |
| Temperature, ^o^C | 38.7 [38.2, 39.4] | 38.4 [38.1, 38.9] |
| Days of fever prior to presentation | 2.5 [1.5, 3.0] | 3.0 [2.0, 3.0] |
| Admission to hospital, number (%) | 6 (20) | 9 (7.2)* |
| *Streptococcus pneumoniae* carriage, number (%) | 28 (93) | 106 (85) |
| ≥1 virus on nasopharyngeal swab, number (%) | 23 (77) | 110 (88) |

^a^ Mann-Whitney U test used for continuous variables, and Chi square test for categorical variables. *, p<0.05. All other comparisons were not significantly different.

^b^ Continuous variables are represented as: Median [Interquartile Range].

^c^ Study sites were located in Dar es Salaam and Ifakara.

^d^ Severe disease defined according to WHO criteria for the district hospital level.

^e^ Participants were subdivided by age, as normal values for respiratory rate are age-dependent [[7](#_ENREF_7)].
