# Supplementary material for: Biomarkers of Host Response Predict Primary End-Point Radiological Pneumonia in Tanzanian Children with Clinical Pneumonia: A Prospective Cohort Study
Source: PLoS One. 2015 Sep 14;10(9):e0137592. doi: 10.1371/journal.pone.0137592 (PMC4569067; doi:10.1371/journal.pone.0137592)
Supplement: S3 Table — (DOCX) [file pone.0137592.s007.docx]

**S3 Table. Receiver operating characteristic (ROC) curves and cut-points of biomarkers that significantly discriminate between clinical pneumonia cases with (n=30) and without (n=125) end-point pneumonia on chest x-ray.^a^**

| **Biomarker** | **AUROCC^b^**  **(95% CI)** | **Cut-point^c^** | **Sensitivity %**  **(95% CI)** | **Specificity %**  **(95% CI)** | **PLR**  **(95% CI)** | **NLR**  **(95% CI)** | **PPV %**  **(95% CI)** | **NPV %**  **(95% CI)** |
| --- | --- | --- | --- | --- | --- | --- | --- | --- |
| **CRP**  (μg/mL) | 0.86  (0.79, 0.93) | >62.2* | 73.3 (54.1-87.7) | 86.4 (79.1-91.9) | 5.4 (3.3-8.8) | 0.31 (0.2-0.6) | 56.4 (39.6-72.2) | 93.1 (86.9-97.0) |
|  |  | >44.1 | 80.0 (61.4-92.3) | 77.6 (69.3-84.6) | 3.6 (2.5-5.2) | 0.26 (0.1-0.5) | 46.2 (32.1-60.7) | 94.2 (87.7-97.8) |
|  |  | >29.8 | 86.7 (69.3-96.2) | 70.4 (61.6-78.2) | 2.9 (2.2-4.0) | 0.19 (0.08-0.5) | 41.3 (29.0-54.4) | 95.7 (89.2-98.8) |
|  |  | >24.3 | 90.0 (73.5-97.9) | 60.8 (51.7-69.4) | 2.3 (1.8-2.9) | 0.16 (0.06-0.5) | 35.5 (24.9-47.3) | 96.2 (89.3-99.2) |
| **PCT**  (ng/mL) | 0.65  (0.53, 0.77) | >2.2* | 40.0 (22.7-59.4) | 89.6 (82.9-94.3) | 3.9 (2.0-7.6) | 0.67 (0.5-0.9) | 48.0 (27.4-69.1) | 86.2 (79.0-91.6) |
|  |  | >0.32 | 80.0 (61.4-92.3) | 34.4 (26.1-43.4) | 1.2 (1.0-1.5) | 0.58 (0.3-1.2) | 22.6 (15.1-31.8) | 87.8 (75.2-95.4) |
|  |  | >0.28 | 86.7 (69.3-96.2) | 28.8 (21.1-37.6) | 1.2 (1.0-1.5) | 0.46 (0.2-1.2) | 22.6 (15.3-31.4) | 90.0 (76.1-97.3) |
| **CHI3L1**  (ng/mL) | 0.79  (0.71, 0.88) | >70.6 | 80.0 (61.4-92.3) | 73.6 (65.0-81.1) | 3.0 (2.2-4.3) | 0.27 (0.1-0.6) | 42.1 (29.1-55.9) | 93.9 (87.1-97.7) |
|  |  | >59.6 | 86.7 (69.3-96.2) | 68.8 (59.9-76.8) | 2.8 (2.1-3.7) | 0.19 (0.08-0.5) | 40.0 (28.0-52.9) | 95.6 (89.0-98.8) |
|  |  | >56.7* | 93.3 (77.9-99.2) | 66.4 (57.4-74.6) | 2.8 (2.1-3.6) | 0.10 (0.003-0.4) | 40.0 (28.5-52.4) | 97.6 (91.8-99.7) |

Abbreviations: AUROCC, area under receiver operating characteristic curve; CHI3L1, Chitinase 3-like-1; CRP, C-reactive protein; CXR, chest x-ray; NLR, negative likelihood ratio; NPV, negative predictive value; PCT, procalcitonin; PLR, positive likelihood ratio; PPV, positive predictive value; ROC, receiver operating characteristic curve; sTie-2, soluble Tie-2; vWF, von Willebrand Factor.

^a^ Children with clinical pneumonia were categorized based on the presence or absence of end-point radiological pneumonia on chest x-ray. Children without end-point pneumonia had either normal CXR or other infiltrates.

^b^ AUROCC p values were <0.001 for CRP and CHI3L1, and 0.012 for PCT.

^c^ Cut-points marked with an asterisk are based on the Youden index: J = max(sensitivity + specificity – 1).
